# Clinical improvements in temporospatial gait variables after a spinal tap test in individuals with idiopathic normal pressure hydrocephalus

**DOI:** 10.1038/s41598-024-52516-3

**Published:** 2024-01-24

**Authors:** Sunee Bovonsunthonchai, Theerapol Witthiwej, Roongtiwa Vachalathiti, Pichaya Hengsomboon, Suthasinee Thong-On, Sith Sathornsumetee, Chanon Ngamsombat, Orasa Chawalparit, Weerasak Muangpaisan, Jim Richards

**Affiliations:** 1 Faculty of Physical Therapy, Mahidol University, Nakhon Pathom, Thailand; 2 Division of Neurosurgery, Department of Surgery, Faculty of Medicine Siriraj Hospital, Mahidol University, Bangkok, Thailand; 3 Division of Neurology, Department of Medicine, Faculty of Medicine Siriraj Hospital, Mahidol University, Bangkok, Thailand; 4 Faculty of Medicine Siriraj Hospital, NANOTEC-Mahidol University Center of Excellence in Nanotechnology for Cancer Diagnosis and Treatment, Mahidol University, Bangkok, Thailand; 5 Department of Radiology, Faculty of Medicine Siriraj Hospital, Mahidol University, Bangkok, Thailand; 6 Department of Preventive and Social Medicine, Faculty of Medicine Siriraj Hospital, Mahidol University, Bangkok, Thailand; 7 Allied Health Research Unit, University of Central Lancashire, Preston, UK

**Keywords:** Hydrocephalus, Disability

## Abstract

Idiopathic Normal Pressure Hydrocephalus (iNPH) is a neurological condition that often presents gait disturbance in the early stages of the disease and affects other motor activities. This study investigated changes in temporospatial gait variables after cerebrospinal fluid (CSF) removal using a spinal tap test in individuals with idiopathic normal pressure hydrocephalus (iNPH), and explored if the tap test responders and non-responders could be clinically identified from temporospatial gait variables. Sixty-two individuals with iNPH were recruited from an outpatient clinic, eleven were excluded, leaving a total of 51 who were included in the analysis. Temporospatial gait variables at self-selected speed were recorded at pre- and 24-h post-tap tests which were compared using Paired t-tests, Cohen’s *d* effect size, and percentage change. A previously defined minimal clinical important change (MCIC) for gait speed was used to determine the changes and to classify tap test responders and non-responders. A mixed model ANOVA was used to determine the within-group, between-group, and interaction effects. Comparisons of the data between pre- and post-tap tests showed significant improvements with small to medium effect sizes for left step length, right step time, stride length and time, cadence, and gait speed. Gait speed showed the largest percentage change among temporospatial gait variables. Within-group and interaction effects were found in some variables but no between-group effect was found. Tap test responders showed significant improvements in right step length and time, stride length and time, cadence, and gait speed while non-responders did not. Some individuals with iNPH showed clinically important improvements in temporospatial gait variables after the tap test, particularly in step/stride length and time, cadence, who could be classified by gait speed. However, gait-related balance variables did not change. Therefore, additional treatments should focus on improving such variables.

## Introduction

Idiopathic normal pressure hydrocephalus (iNPH) is a neurological condition characterized by ventriculomegaly and normal cerebrospinal fluid (CSF) pressure, was first described by Hakim and Adams in 1965^1^.The three cardinal features comprised gait disturbance, cognitive impairment, and urinary incontinence^2–4^. The iNPH is considered a potentially treatable neurological disorder if diagnosed in a timely manner and properly managed^2,5,6^. Diagnosis of iNPH is based on medical history, physical examination, and brain imaging with Computerized Tomography (CT) or Magnetic Resonance Imaging (MRI)^7^. CSF removal with shunt surgery is the only known effective treatment method with around 60–80% of patients reported to improve^7^. So, a preliminary evaluation is therefore essential due to surgery being an invasive method that requires cost and care of further complications, especially in the older population. Different evaluation methods such as extended lumbar drainage, infusion test, intracranial pressure monitoring, and tap test have been investigated and used to predict the response to shunt surgery for individuals with iNPH^8^.

In general, altered gait is a manifestation of the early symptoms of disease accompanied by motor function impairments and increased risk of falls, whereas cognitive impairment and urinary incontinence may appear later^9^. The best way to describe gait impairment of iNPH is as a higher-level gait disorder, which is the absence of primary sensorimotor deficits, cerebellar dysfunction, or involuntary movements^10^. This impairment is often symmetrical unless there is a coexisting abnormality of the musculoskeletal system, causing an imbalance. Problems with gait initiation, shuffling, festination, inadequate foot clearance, tripping, falling, sit-to-stand difficulties, and unstable multiple-step turns can occur in individuals with iNPH^10–14^. Different quantitative gait variables such as temporo-spatial, kinetics, and kinematics have been studied^15,16^. Among the three cardinal features, the easiest and most effective outcome to assess the tap test response is gait disturbance^17^ which was used to predict shunt surgery responsiveness in individuals with iNPH^15,18,19^. Gait improvement could be seen immediately or within a few days after the tap test^20,21^. Gait speed was the most responsive variable to the tap test, followed by cadence, step length, en bloc turning, and step height^21^.

Compared to healthy controls, individuals with iNPH have been shown to exhibit a slower speed, shorter stride length, broad base of support, increased percentage of stance phase and double limb support, increased step number, step time, and decreased cadence^13,22,23^. They also had a greater variability of step time and stride length^22,23^. These alterations of gait parameters can be improved after the tap test for the responders^13,23^. However, a controversial result was reported in a recent pilot study of eight individuals with iNPH and found no change in their gait^24^.

Until present, the positive and negative responsiveness criteria to the CSF tap test are varied among studies^3,13,22,25–27^. The proposed criteria include; observation of clinical symptoms^13,25^, self-report from the patients or caregivers^13,26^, or an improvement in the iNPH grading scale of 1 score or an improvement of more than 5‒10% of change in the Timed Up and Go or gait speed using the 10-m walk test^3,22,27^. In addition, a complex classification criterion with a reduction of at least 10% in Mahalanobis distance was used in another study^28^. However, these established change thresholds may be limited as they do not reflect an important actual change in clinical practice or are too difficult to use in a real-world situation. Therefore, the purpose of this study was to examine the changes in temporospatial gait variables in individuals with iNPH and to compare these outcomes between tap test responders and non-responders based on a minimal clinical important change (MCIC). Gait speed has been used as an important determinant of health in the elderly and evidence suggests that gait speed may be able to predict several adverse outcomes^29,30^ and could be an indicator of post-tap status for individuals with iNPH^10,20,22^. We, therefore, hypothesized that temporospatial gait variables would be improved with gait speed being the most sensitive variable to detect tap test responders from non-responders to CSF removal using a tap test in individuals with iNPH.

## Materials and methods

### Study design and recruitment

A single group with a pre-and post-test design was used in this study. Individuals with iNPH were recruited from the outpatient department clinic, Siriraj Hospital, Thailand. They were initially screened by neurologists and neurosurgeons. Individuals with iNPH and their caregivers were informed about the research objectives, possible benefits, and details by the research team. If they agreed to participate and signed a consent form an appointment for a hospital admission and time for gait assessment were booked.

### Participants

Sixty-two individuals with possible iNPH were recruited in this study. They were diagnosed by neurologists or neurosurgeons following the Japanese Society of iNPH management guidelines^25^. Inclusion criteria were; more than one of the symptoms of gait disturbance, cognitive impairment, and urinary incontinence, 60 years of age or older, ventricle enlargement assessed by MRI, CSF pressure of 200 mmH_2_O or lower with normal CSF contents. They were excluded if unable to undergo a spinal tap test, unable to MRI, and had other conditions that caused an inability to assess gait. A tap test with CSF removal of 30–50 cc was performed by the neurosurgeon. Eleven individuals with iNPH were excluded as they were unable to perform the walk test (n = 4), exhausted (n = 1), herpes zoster (n = 1), unable to communicate or follow commands (n = 2), gout (n = 1), severe back pain (n = 1), and incomplete data (n = 1). Therefore, a total of 51 individuals with iNPH completed the assessment and were used in the analysis and the presence of Disproportionately Enlarged Subarachnoid Space Hydrocephalus (DESH) was reviewed by an experienced neurosurgeon on the MRI images.

### Ethical consideration

This research complied with the institutional policy and followed the tenets of the Declaration of Helsinki. All participants were informed about the research details and signed the informed consent approved by the Siriraj Institutional Review Board (COA no: SI 340/2014). Data were collected from 2 February 2015 to 10 December 2017 at the Division of Neurology, Department of Medicine, Faculty of Medicine, Siriraj Hospital, Thailand.

### Procedure

Temporo-spatial gait parameters were recorded at pre- and 24-h post-tap tests using an objective gait measurement platform [Force Distribution Measurement (FDM), Zebris Medical GmbH, Germany] at the participants most comfortable speed. A 3 m walking platform was installed in the middle part of a 5 m walkway and was synchronized with a video camera (SYNCCam) placed at the end of the walkway. Gait data were recorded at a sample frequency of 100 Hz. The gait measurement platform has been previously used to assess older adults and many neurological cases^31–33^ and was used as a reference standard^34^.

To obtain data accurately, the same instructions and demonstration were used before data collection. Participants were instructed to walk when they saw a light signal and were asked to “start” walking. A physical therapist walked behind individuals with iNPH to provide care or assistance as needed but did not directly interfere with their walk. Gait data were collected for 3 trials and the averaged data were used in the analysis. To reduce any acceleration or deceleration effects, data from the middle part of the gait mat was selected and used in the analysis using the Win-FDM Software (Zebris Medical GmbH, Germany, version 1.18.48).

### Outcome measures

Temporo-spatial gait variables included the left and right foot rotation angle (deg), step length (cm), percentage of stance phase of one gait cycle (%GC), loading response (%GC), single limb support (%GC), pre-swing (%GC), swing phase (%GC), step time (s), stride length (cm), step width (cm), double limb support (%GC), stride time (s), cadence (steps/min), gait speed (m/s).

### Minimum clinical important change (MCIC) criteria and definition of tap test responders and non-responders

Gait speed has been considered a major indicator of post-tap test outcomes^10,20,22^. Although the minimum clinically important change (MCIC) criteria have been reported to be varied in people with different pathologies^35^, no previous study has reported a MCIC for gait speed after tap test or shunt surgery in individuals with iNPH. Previously a MCIC criteria for gait speed was reported in patients with Parkinson’s disease by Hass et al.^36^, which determined a small important difference in gait speed of 0.06 m/s, a moderate important difference of 0.14 m/s, and a large important difference of 0.22 m/s. Therefore individuals with iNPH were categorized as tap test responders using 0.06 m/s or greater, or non-responders less than 0.06 m/s.

### Statistical analysis

Data were analyzed using SPSS software version 23.00 (IBM Corp, USA). The Shapiro–Wilk test was used to test data distribution and data were found to be suitable for parametric testing. To reduce type I error from the multiple hypotheses tested in this study, we used the Bonferroni correction method^37,38^ with the set p-value of 0.05/number of hypotheses tested (k = 22). So, the adjusted alpha value was *p* < 0.05/22 or *p* < 0.0023. Grouped data were compared between the pre- and post-tap test and the effect sizes were determined using Cohen’s *d*^39^. The guideline for the interpretation of effect size values with small, medium, and large are 0.10, 0.30, and 0.50, respectively^40^.

Participants were then classified into tap test responders and non-responders and a two-way mixed model ANOVA was used to quantify the within-group effect (pre- and post-tap tests), between-group effect (tap test responders and non-responders), and their interactions. Partial Eta Square (η_p_^2^) was used to determine the effect size with the interpretation of small (0.01), medium (0.06), and large (0.14). In case the interaction effects were seen, sub-analysis comparing the data between the pre- and post-tap tests for each group and between groups at each time point were tested.

## Results

### Participant characteristics

Fifty-one individuals with iNPH with 31 males and 20 females completed the study. Their average age was 78.3 ± 6.3 years, weight 57.1 ± 11.3 kg, and height 158.8 ± 9.9 cm. The average duration of the disease was 18.1 ± 23.6 months, with fourteen of the participants assessed as having DESH and the rest non-DESH (n = 37). The majority of them had problems with gait (n = 46), followed by cognitive impairment (n = 41), and urinary incontinence (n = 35). Most of them could walk without using any gait aids (n = 32). A small number reported using a cane (n = 10) or walker (n = 9) at home occasionally. During the gait assessment, most of the participants did not need assistance but some needed it, with 13 requiring mild and 10 requiring moderate assistance. For the underlying disease, nearly half of them had hypertension (n = 23), followed by dyslipidemia (n = 15) and diabetes mellitus (n = 13). In addition, some of them had heart disease (n = 8), stroke (n = 8), Parkinson’s disease (n = 4), and chronic kidney disease (n = 3).

### Effect of the tap-test on temporospatial gait variables

Comparisons of the temporospatial gait variables between pre- and post-tap tests are presented in Table 1. Significant differences (*p* < 0.0023) were shown in left step length, right step time, stride length and time, cadence, and gait speed while the remaining variables showed no differences (p > 0.0023). A small effect size (*d* = 0.1) was found in left step length, stride length and time, and gait speed. In addition, the right step time and cadence showed a medium effect size (*d* = 0.3). The percentage change was calculated from the difference between the mean scores of the pre-and post-tap tests for data showing significance. It was found that the largest change in temporo-spatial gait variables after the tap test was gait speed with a 10.5% improvement.

**Table 1:** Comparisons of the temporospatial gait variables between pre- and post-tap tests (n = 51).

Variables	Pre-tap mean ± SD	Post-tap mean ± SD	p-value^a^	Effect size^b^	% of change
Foot rotation angle—Left (deg)	18.74 ± 9.63	17.72 ± 9.72	0.075	0.11	‒
Foot rotation angle—Right (deg)	18.07 ± 9.03	18.80 ± 8.66	0.189	− 0.08	‒
Step length—Left (cm)	23.92 ± 8.71	25.77 ± 9.17	**0.001**	− **0.21**	**7.73**
Step length—Right (cm)	24.06 ± 8.80	25.54 ± 10.20	0.020	− 0.15	6.15
Stride length (cm)	47.94 ± 16.81	51.23 ± 18.27	** < 0.001**	− **0.18**	**6.86**
Step width (cm)	17.08 ± 4.00	17.27 ± 3.77	0.492	− 0.05	‒
Stance phase—Left (%GC)	78.27 ± 7.00	78.29 ± 6.62	0.973	− 0.00	‒
Stance phase—Right (%GC)	77.64 ± 6.89	77.32 ± 7.27	0.602	0.04	‒
Load response—Left (%GC)	28.31 ± 7.21	27.66 ± 7.06	0.307	0.09	‒
Load response—Right (%GC)	27.27 ± 6.96	27.84 ± 6.29	0.378	− 0.09	‒
Single limb support—Left (%GC)	22.59 ± 6.93	22.96 ± 7.14	0.550	− 0.05	‒
Single limb support—Right (%GC)	22.06 ± 7.28	21.74 ± 6.71	0.641	0.05	‒
Pre-swing—Left (%GC)	27.24 ± 6.69	27.55 ± 6.17	0.613	− 0.05	‒
Pre-swing—Right (%GC)	28.38 ± 7.30	27.64 ± 7.00	0.225	0.10	‒
Swing phase—Left (%GC)	21.73 ± 7.00	21.71 ± 6.62	0.973	0.00	‒
Swing phase—Right (%GC)	22.36 ± 6.89	22.68 ± 7.27	0.602	− 0.04	‒
Double limb support (%GC)	55.74 ± 12.95	55.51 ± 12.61	0.817	0.02	‒
Step time—Left (s)	0.71 ± 0.29	0.67 ± 0.28	0.004	0.12	4.90
Step time—Right (s)	0.69 ± 0.16	0.64 ± 0.13	**0.001**	**0.33**	**6.96**
Stride time (s)	1.36 ± 0.31	1.28 ± 0.27	**0.001**	**0.28**	**6.12**
Cadence (steps/min)	92.26 ± 17.42	97.63 ± 17.45	** < 0.001**	− **0.31**	**5.82**
Gait speed (m/s)	0.38 ± 0.16	0.42 ± 0.17	** < 0.001**	− **0.26**	**10.53**

^a^p-value tested by the Paired *t*-test at < 0.0023; Significance shown in bold values.

^b^Effect size for the Paired *t*-test (Cohen’s *d*).

### Changes in gait speed compared to the minimal clinically important change (MCIC)

From a total of 51 participants, 35 subjectively reported walking more easily or faster, while 10 felt the same and 6 felt slightly diminished. When comparing gait speed obtained from the objective method between pre-and post-tap tests of individuals with the set clinical change criteria, 23 from 51 had an improvement (45.1%). Of this number, 19 (37.3%) reached the threshold for a small clinical improvement, and 4 (7.8%) reached the threshold for a moderate clinical improvement in gait speed. Whereas 25 (49.0%) had no change and 3 (5.9%) had deteriorated (Table 2).

**Table 2:** Number and proportions of individuals with iNPH who had improved or deteriorated in gait speed according to the threshold for a Minimal Clinically Important Change (MCIC) (n = 51).

Criteria		Gait speed (m/s)	Total
Improved n (%)	Small (+ 0.06 to 0.13 m/s)	19 (37.3%)	23 (45.1%)
	Moderate (+ 0.14 to 0.21 m/s)	4 (7.8%)	
	Large (≥ + 0.22 m/s)	0 (0%)	
No change n (%)	(+ /- < 0.06 m/s)	25 (49.0%)	25 (49.0%)
Deteriorated n (%)	(> − 0.06 m/s)	3 (5.9%)	3 (5.9%)
	**51 (100%)**		

Table 3 shows comparisons of the characteristics between tap test responders and non-responders. There was no significant difference in all testing variables, except for walking assisted by physical therapists (p = 0.039). It was found that a greater number of tap test non-responders required assistance while walking than responders.

**Table 3:** Characteristics of tap test responders and non-responders.

Variables	Responders (n = 23)	Non-responders (n = 28)	p-value
Age (years), mean ± SD	77.35 ± 7.07	79.07 ± 5.60	0.336^a^
Weight (kg), mean ± SD	60.39 ± 10.54	54.36 ± 11.30	0.056^a^
Height (cm), mean ± SD	159.04 ± 11.45	158.61 ± 8.74	0.878^a^
Disease duration (months), mean ± SD	17.24 ± 23.22^α^	18.84 ± 24.26^β^	0.818^a^
Disproportionately enlarged subarachnoid space hydrocephalus (DESH), n (%)			
DESH	8 (34.78)	6 (21.43)	0.353^b^
Non-DESH	15 (65.22)	22 (78.57)	
Sex, n (%)			
Male	15 (65.22)	12 (42.86)	0.557^b^
Female	8 (34.78)	16 (57.14)	
Dominant side, n (%)			
Left	0 (0)	0 (0)	‒
Right	23 (100)	28 (100)	
Clinical symptoms^γ^, n (%)			
Gait disturbance	20 (86.96)	26 (92.86)	0.647^c^
Cognitive impairment	18 (78.26)	23 (82.14)	0.739^c^
Urinary incontinence	17 (73.91)	18 (64.29)	0.461^b^
Usual gait aid, n (%)			
None	17 (73.91)	15 (53.57)	0.068^d^
Cane	5 (21.74)	5 (17.86)	
Walker	1 (4.35)	8 (28.57)	
Walking assisted by a physical therapist, n (%)			
None	16 (69.57)	12 (42.86)	**0.039**^d^
Mild	5 (21.74)	8 (28.57)	
Moderate	2 (8.70)	8 (28.57)	
Underlying diseases^γ^, n (%)			
Hypertension	12 (52.17)	11 (39.29)	0.357^b^
Dyslipidemia	8 (34.78)	7 (25.00)	0.446^b^
Diabetes Mellitus	4 (17.39)	9 (32.14)	0.229^b^
Heart disease	5 (21.74)	2 (7.14)	0.221^c^
Stroke	2 (8.70)	6 (21.43)	0.269^c^
Parkinson’s disease	1 (4.35)	5 (17.86)	0.362^c^
Chronic kidney disease	2 (8.70)	1 (3.57)	0.583^c^

Missing data from 2 tap test responders^α^ and 1 non-responder^β^ due to no onset data being available; ^γ^Participants able to report more than one problem, ^a^p-value tested using the Independent Sample t-test ^b^p-value tested using the Chi-Square test; ^c^p-value tested using the Fisher Exact test; ^d^p-value tested using the Mann–Whitney U test at *p* < 0.05.

### Comparisons of the data between tap test responders and non-responders

Table 4 shows a two-way mixed model ANOVA for the temporospatial gait variables. Within-group effects were found in left and right step lengths, left and right step times, stride length and time, cadence, and gait speed. Interaction effects were found in the right step length and time, stride length and time, cadence, and gait speed. However, no between-group effect was found in any variables.

**Table 4:** Two-way mixed model ANOVA for the temporospatial gait variables.

Variables	Within-group effect (pre- and post-tap tests)			Between-group effect (responders and non-responders)			Interaction effect	
	Mean diff	*p *value^a^	η_p_^2^	Mean diff	*p *value^a^	η_p_^2^	*p *value^a^	η_p_^2^
Foot rotation angle—Left (deg)	0.943	0.098	0.055	− 4.946	0.063	0.069	0.143	0.043
Foot rotation angle—Right (deg)	− 0.651	0.237	0.028	− 1.290	0.600	0.006	0.145	0.043
Step length—Left (cm)	− 1.999	** < 0.001**	0.253	2.665	0.283	0.023	0.004	0.158
Step length—Right (cm)	− 1.747	**0.001**	0.209	4.210	0.107	0.052	** < 0.001**	0.390
Stride length (cm)	− 3.715	** < 0.001**	0.393	6.996	0.152	0.041	** < 0.001**	0.457
Step width (cm)	− 0.210	0.460	0.011	− 0.130	0.904	< 0.001	0.539	0.008
Stance phase—Left (%GC)	0.086	0.886	< 0.001	− 3.008	0.098	0.055	0.074	0.064
Stance phase—Right (%GC)	0.417	0.484	0.010	− 1.258	0.513	0.009	0.086	0.059
Load response—Left (%GC)	0.770	0.210	0.032	− 1.466	0.448	0.012	0.040	0.083
Load response—Right (%GC)	− 0.479	0.454	0.012	− 2.348	0.183	0.036	0.164	0.039
Single limb support—Left (%GC)	− 0.466	0.579	0.450	0.756	0.692	0.003	0.123	0.048
Single limb support—Right (%GC)	0.198	0.766	0.002	2.570	0.167	0.039	0.075	0.063
Pre-swing—Left (%GC)	− 0.239	0.700	0.003	− 2.389	0.163	0.039	0.222	0.030
Pre-swing—Right (%GC)	0.871	0.146	0.043	− 1.498	0.441	0.012	0.036	0.087
Swing phase—Left (%GC)	− 0.087	0.886	< 0.001	3.009	0.098	0.055	0.074	0.064
Swing phase—Right (%GC)	− 0.417	0.484	0.010	1.258	0.513	0.009	0.086	0.059
Double limb support (%GC)	0.417	0.661	0.004	− 3.888	0.266	0.025	0.045	0.080
Step time—Left (s)	0.038	**0.001**	0.195	− 0.110	0.167	0.039	0.008	0.136
Step time—Right (s)	0.052	** < 0.001**	0.260	− 0.053	0.168	0.038	**0.002**	0.178
Stride time (s)	0.090	** < 0.001**	0.262	− 0.101	0.193	0.034	**0.002**	0.185
Cadence (steps/min)	− 5.800	** < 0.001**	0.443	6.930	0.149	0.042	** < 0.001**	0.312
Gait speed (m/s)	− 0.050	** < 0.001**	0.618	0.080	0.078	0.062	** < 0.001**	0.647

^a^*p*-value tested by the two-way mixed ANOVA at < 0.0023; Significance shows in bold values; Mean diff = Mean difference; η_p_^2^ = Partial Eta Square.

Within-group effects were found in the sub-analyses between pre- and post-tap test for responder and non-responder groups. Table 5 shows sub-analyses of the temporospatial gait variables between pre- and post-tap tests for each group of tap test responders and non-responders, with significant improvements in right step length and time, stride length and time, cadence, and gait speed being seen in the tap test responders only.

**Table 5:** Sub-analyses of the temporospatial gait variables between pre- and post-tap tests for tap test responders and non-responders where an interaction effect was seen.

Variables	Responders (n = 23)			Non-responders (n = 28)		
	Pre-tap Mean ± SD	Post-tap Mean ± SD	p-value^a^	Pre-tap Mean ± SD	Post-tap Mean ± SD	p-value^b^
Step length—Right (cm)	24.88 ± 8.89	29.35 ± 9.56	** < 0.001**	23.39 ± 8.83	22.42 ± 9.78	0.200
Stride length (cm)	49.45 ± 17.83	57.40 ± 19.07	** < 0.001**	46.69 ± 16.15	46.17 ± 16.20	0.577
Step time—Right (s)	0.68 ± 0.17	0.59 ± 0.09	** < 0.001**	0.70 ± 0.15	0.69 ± 0.15	0.439
Stride time (s)	1.34 ± 0.34	1.18 ± 0.19	** < 0.001**	1.37 ± 0.29	1.36 ± 0.30	0.462
Cadence (steps/min)	93.66 ± 17.62	103.84 ± 14.69	** < 0.001**	91.11 ± 17.48	92.53 ± 18.11	0.262
Gait speed (m/s)	0.39 ± 0.16	0.49 ± 0.17	** < 0.001**	0.36 ± 0.16	0.36 ± 0.15	0.675

^a^Comparison between pre- and post-tap tests in tap test responders by the Paired t-test.

^b^Comparison between pre- and post-tap tests in tap test non-responders by the Paired t-test.

## Discussion

### Participants characteristics

The mean age of all participants was 78 years, which was similar to previous studies that reported the incidence of iNPH commly found in the elderly^41,42^. The mean disease duration of our participants was 18 months. Disease duration was reported to be associated with the accuracy of the tap test in predicting surgical success^18^ and could affect shunt surgery responsiveness^43,44^. Similar to previous reports, most of the participants of this study were male with approximately 61%^41,42,45^. The predisposition of males to iNPH over females may be explained by pathophysiological differences. Males have greater CSF stroke rates and aqueductal flow volumes while females have more brain viscoelasticity^45^

For clinical triad, gait disturbance was the most frequent, followed by cognitive deficits and urinary incontinence which is consistent with previous studies^41,42^. For walking aid usage, most participants could walk independently, with only a small number of tap test responders needing to use a cane, while some of non-responders needed to use a walker. Most of them were able to walk on their own without any help from a physical therapist. The most common comorbidity for individuals with iNPH in this study was hypertension, consistent with previous reports^42,46,47^. It was reported that hypertension may be involved in mechanisms promoting iNPH pathogenesis^48^ and was one of the predictors of unfavorable outcomes and independent walking after shunt surgery^44^.

### Effect of the tap test on temporospatial gait variables

Comparisons of the temporospatial gait variables between pre- and post-tap tests in the whole group of participants showed that CSF removal by tap test had some gait benefits and improved left step length, right step time, stride length and time, cadence, and gait speed, which was consistent with previous reports that showed improvements in stride length^10,22,49^ and gait speed^10,22,28,49^. The results of this study differed from previously reported studies which showed improvement in step width^22^ but our study did not. However, it should be noted that an increase in step width was merely 0.67 cm, representing a 2.6% improvement^22^. In our study, the temporo-spatial gait improvement ranged between small to moderate effects and the percentage of improvement was between 4.9 and 10.5% showing gait speed had the greatest percentage of change. Regarding the effect size and percentage of change, these are very helpful in considering the magnitude of change, whereas statistical significance examines whether the findings are likely to be due to chance^50^.

For the other temporospatial gait variables; foot rotation angle and step width, which were considered as specific features of gait disturbance for individuals with iNPH^51^, showed no improvement after the tap test in this study. In addition, no changes in the stance phase, load response, single limb support, preswing, swing phase, and double limb support variables were found in this study. All of which were related to the ability of postural control and balance during walking. This may imply that individuals with iNPH had significant problems with postural control and balance, which can affect confidence and risk of falling. It might be possible to imply that these gait-related balance variables could not be improved by the tap test.

### Changes in gait speed compared to the minimal clinically important change (MCIC)

Considering the MCIC threshold for change in gait speed, the present study found that 45% of participants had improvements with small to moderate effects, 49% had no change, and 6% of participants gait speed deteriorated after the tap test. As gait disorders in individuals with iNPH and Parkinson’s disease share the same abnormal feature of a reduced gait speed^51^ and as mentioned in a recently published report that no MCIC of gait speed after tap test or shunt surgery has previously been reported in individuals with iNPH^52^, the MCIC criteria used in this study was taken from a previous study that was conducted on 324 ambulatory patients with Parkinson’s disease^36^. In the present study, a clinical improvement in gait speed with a small effect was found in most (37.3%), a moderate effect was found in some (7.8%), and no large effect was found in individuals with iNPH who responded to the intervention. These different response levels in the tap test responders may be related to the duration of the assessment^53^ and may be important in predicting shunt surgery success in the future. However, because of these MCIC criteria, the number of individuals who responded to the intervention was less than half (45.1%), unlike the results reported in a previous study where people who had a positive response ranged from 67‒86**%** of participants^19,46,54^. Differences in the results between studies may be caused by many reasons, such as different criteria used for tap test responders or non-responders, severity and duration of the disease, assessment time, etc.

### Comparison between Tap test responders and non-responders

For within-group comparison, tap test responders showed significant improvements in right step length and time, stride length and time, cadence, and gait speed. In contrast, the tap test non-responders showed no significant improvements in all tested variables. This is consistent with previous studies which reported increases in stride length^13,19,51^, cadence^13^, and gait speed^13,19,54^ in the tap test responders. However, previous studies found an increase in double limb support^13,19^, which was not seen in this current study. When considering the variables which improved for tap test responders, the mean values remained far from the elder’s norm values reported in a previous study^31^. This may support the consideration of additional treatment, such as surgery and specific therapeutic exercise to develop the gait ability and a longer-term follow-up is also needed.

In addition to the reduced gait speed and stride length that are commonly seen, the presence of broad-based with outward rotated feet has been also often defined as abnormal gait features in individuals with iNPH^22,51,54^. Other gait-related balance variables that were reported over a percentage of a gait cycle, such as stance phase, single limb support, swing phase, and double limb support were not improved. All of these confirmed that those insensitive variables were likely to be problematic for individuals with iNPH and difficult to recover after 24 h of the CSF tap test, even when assessed among the tap test responders. Therefore, for these insensitive variables, it may be a challenge to devise additional therapeutic approaches after shunt surgery, to develop dynamic balance skills.

This study may be limited by the lack of healthy controls to compare the data with individuals with iNPH, and a larger sample size with a longer follow up assessment time are needed to provide a more robust view of the effectiveness of the tap test. The MCIC criteria used to classify individuals on gait speed were based on values reported in patients with Parkinson's disease who have similar impairments. However this value has not been previously reported in iNPH, and the number of individuals responding to the tap test might be different with a specific MCIC for iNPH. In our study, individuals with iNPH who could not walk were excluded, resulting in the findings not covering all levels of disease severity. The strength of this study is to provide quantitative gait analysis after the CSF tap test using a MCIC cut-off threshold, and the quantitative test provides robust information that should be considered alongside subjective clinical assessments, and patients and caregivers' opinions.

## Conclusion

Individuals with iNPH enhanced their gait after the tap test as evidenced by the improvement of step length, stride length, step time, stride time, cadence, and gait speed which provide robust information that should be considered alongside subjective clinical assessments. However, other gait-related balance variables; foot rotation angle, step width, stance phase, load response, single limb support, pre-swing, swing phase, and double limb support did not change. Therefore, additional treatments for gait-related balance ability are needed to improve gait performance.

## Data Availability

The data are available from the authors upon reasonable request.

## Abbreviations

iNPH: Idiopathic normal pressure hydrocephalus
CSF: Cerebrospinal fluid
CT: Computerized tomography
MRI: Magnetic resonance imaging
MCIC: Minimal clinical important change
DESH: Disproportionately enlarged subarachnoid space hydrocephalus
